# The mitochondrial genome and phylogenetic characteristics of the Thick-billed Green-Pigeon, *Treron curvirostra*: the first sequence for the genus

**DOI:** 10.3897/zookeys.1041.60150

**Published:** 2021-06-02

**Authors:** Nan Xu, Jiayu Ding, Ziting Que, Wei Xu, Wentao Ye, Hongyi Liu

**Affiliations:** 1 College of Biology and the Environment, Nanjing Forestry University, Nanjing 210037, China Nanjing Forestry University Nanjing China

**Keywords:** Columbidae, genome sequencing, Ka/Ks ratio, mitochondrial DNA, phylogenetic tree

## Abstract

Members of the genus *Treron* (Columbidae) are widely distributed in southern Asia and the Indo-Malayan Region but their relationships are poorly understood. Better knowledge of the systematic status of this genus may help studies of historical biogeography and taxonomy. The complete mitochondrial genome of *T. curvirostra* was characterized, a first for the genus. It is 17,414 base pairs in length, containing two rRNAs, 22 tRNAs, 13 protein coding genes (PCGs), and one D-loop with a primary structure that is similar to that found in most members of Columbidae. Most PCGs start with the common ATG codon but are terminated by different codons. The highest value of the Ka/Ks ratio within 13 PCGs was found in ATP8 with 0.1937, suggesting that PCGs of the mitochondrial genome tend to be conservative in Columbidae. Moreover, the phylogenetic relationships within Columbidae, which was based on sequences of 13 PCGs, showed that (*T. curvirostra* + *Hemiphaga novaeseelandiae*) were clustered in one clade, suggesting a potentially close relationship between *Treron* and *Hemiphaga.* However, the monophyly of the subfamilies of Columbidae recognized by the Interagency Taxonomic Information System could not be corroborated. Hence, the position of the genus *Treron* in the classification of Columbidae may have to be revised.

## Introduction

Mitochondrial DNA sequences can be reliable markers for studying the origin and phylogenetic relationships of species owing to its fast evolution rate, simple structure, light molecular weight, and maternal inheritance (Nabholz et al. 2016; Martins et al. 2019). Mitochondrial genomes of birds have a closed loop structure with lengths of 15,500–23,000 base pairs (bp) (Sammler et al. 2011; Xu et al. 2019; Wang et al. 2020). They typically contain 13 protein coding genes (PCGs), 22 transfer RNA genes (tRNAs), two ribosomal RNA genes (rRNAs), and one D-loop (Bensch and Härlid 2000; Sun et al. 2020), while some species were found to have duplicate regions (Eberhard and Wright 2016).

The pigeons and doves (family Columbidae) are widely distributed on all continents except Antarctica, ranging from tropical to temperate regions (Gibbs et al. 2001). The number of subfamilies of Columbidae differs among taxonomic authorities. Dickinson and Remsen (2013) recognize three subfamilies (Columbinae, Peristerinae, and Raphinae), whereas the Interagency Taxonomic Information System (ITIS) recognizes five subfamilies (Columbinae, Didunculinae, Gourinae, Otidiphabinae, and Treroninae), as well as 49 genera and more than 300 extant species (Integrated Taxonomic Information System 2020).

All species of green-pigeons (*Treron*) are listed as second-class national protected animals under China’s Catalog of Wildlife of the Key State Protection. Most species in the genus are declining (Birdlife International 2018); however, only a few genetic resources are available for the genus *Treron* (e.g., Sorenson et al. 2003; Pereira et al. 2007; Hackett et al. 2008; Price et al. 2014; Claramunt and Cracraft 2015).

The Thick-billed Green-Pigeon *Treron curvirostra* (Gmelin, 1789) is mainly distributed in virgin, evergreen, broad-leaved, and secondary forests of the tropical and subtropical hilly zone in Southeast Asia and South Asia (Gibbs et al. 2001). Like most species of Columbidae, *T. curvirostra* feeds on seeds and fruits (Korzun et al. 2008). Members of this species have a medium-sized body and a colorful plumage (Korzun et al. 2008) distinguished by their grey head and green neck. The lower body is yellowish green, while the wing is nearly black, with a yellow feather margin and a distinct yellow wing spot. The central tail feathers are green, while the remaining feathers are gray with black secondary end spots (Korzun et al. 2008; Nair 2010). At present, only few studies have focused on *T. curvirostra*: Nair (2010) discussed the zoogeography.

To understand the systematic position of the genus *Treron* among Columbidae, we sequenced and characterized the first complete mitochondrial genome sequence of *T. curvirostra*. We compared the complete mitochondrial genome of *T. curvirostra* with that of 33 other pigeons and doves and determined its genetic structural characteristics. In addition, we used 13 protein-coding genes (PCGs) to reconstruct a phylogenetic tree, which we use to infer the taxonomic position of the species and illuminate the phylogenetic relationships among species of Columbidae.

## Materials and methods

### Sample collection and DNA extraction

This study was authorized by Nanjing Forestry University. The youngest tail feathers of a male Thick-billed Green-Pigeon *T. curvirostra* were collected from an individual rescued from a net that was used to prevent birds from stealing fruit at the Xieyang peak of Dali City, Yunnan Province, China. The bird was identified as *T. curvirostra* based on its morphological characters (Gibbs et al. 2001). After sample collection, the bird was released. The tail feather samples were transported to the Laboratory of Animal Molecular Evolution at the Nanjing Forestry University and stored at -80 °C. The tubules were cut and the pulp was removed for genomic DNA extraction using the FastPure Cell/Tissue Isolation Mini kit (Vazyme Biotechnology Co., Ltd., Nanjing, China) and stored at -20 °C for later use.

### PCR amplification and sequencing

Primers were designed based on the mitochondrial gene sequences of *Streptopelia decaocto*, *Hemiphaga novaeseelandiae*, and *Columba hodgsonii* (GenBank accession numbers KY827036, EU725864, and MN919176,respectively) using DNASTAR software (DNASTAR, USA; Burland 2000). Primer sequences are listed in Table 1. The PCR reaction volume was 25 μL, which included 1 μL of template DNA, 12.5 μL of the 2×Rapid Taq Master Mix (Vazyme Biotech Co., Ltd, Nanjing, China), 1 μL per primer, and 9.5 μL double-distilled (dd)H_2_O. The PCR reaction procedure consisted of a pre-denaturation at 95 °C for 3 min, a denaturation at 95 °C for 15 s, an annealing at 50 °C to 60 °C for 15 s, which was adjusted according to the primers’ own conditions, an extension at 72 °C for 2 min, cycling 35 times, and a final extension at 72 °C for 5 min. The PCR products were detected by a 1% agarose gel electrophoresis, and then sent to Tsingke Biotech Co., Ltd. (Nanjing, China), where the original primers were used for the bidirectional sequencing.

**Table 1.** Primers used for amplification of the *T. curvirostra* mitogenome.

Fragment	Region	Primer pair	Primer sequence (5' - 3')
DG 1	COI-COII	DG 1F	CACTCAGCCATCTTACCT
		DG 1R	ACAGATTTCTGAGCATTGGC
DG 2	COII-ND4	DG 2F	CCAATCCGCATCATCGTC
		DG 2R	GGTTTCCTCATCGTGTGA
DG 3	ND4-ND5	DG 3F	CAGCCTCCTAATTGCCAC
		DG 3R	GTAGGGCGGAGACTGGAG
DG 4	ND5-Cyt b	DG 4F	ACAGGGCCGAGCAGAAGC
		DG 4R	TAGGAAGTATCACTCTGG
DG 5	Cyt b-12S rRNA	DG 5F	GCAGGCCTCACCATTATCC
		DG 5R	GTTAATTACTGCTGAGTACC
DG 6	12S rRNA-16S rRNA	DG 6F	GCTGGCATCAGGCACGCC
		DG 6R	TGGGTCTGGTTACTGTTA
DG 7	16S rRNA-ND2	DG 7F	CGGTTGGGGCGACCTTGGAG
		DG 7R	AGAGTGGGAGGAGTAGGGC
DG 8	ND2-COI	DG 8F	AGCAGCCACAATCATGGC
		DG 8R	ATAGATTTGGTCATCTCC

### Sequence analysis

By comparing and identifying the DNA sequence of each mitochondrial gene in other pigeon families, the range and location of *T. curvirostra*’s mitochondrial genes were annotated. Hence, the complete mitochondrial genome sequence was used to predict the transcriptional direction of each gene component using the Improved de novo Metazoan Mitochondrial Genome Annotation (MITOS) platform (Bernt et al. 2013). The annotated mitochondrial genome sequence of *T. curvirostra* was submitted to GenBank (accession number MT535857). The mitochondrial ring structure was plotted, and 22 tRNA clover two-dimensional structures were predicted using programs, such as the comparative genomics (CG) View Server and the tRNAscan-Se (Stothard and Wishart 2005; Lowe and Chan 2016). Composition skew was calculated according to the following formulae: AT-skew = (A-T)/(A+T) and GC-skew = (G-C)/(G+C) (Perna and Kocher 1995). Moreover, the relative synonymous codon usage (RSCU) frequency and the ratio of the number of nonsynonymous substitutions per nonsynonymous site to the number of synonymous substitutions per synonymous site (Ka/Ks) of 13 PCGs of Columbidae were calculated using MEGA7 (Kumar et al. 2016), while the RSCU comparison graph was drawn by PhyloSuite (Zhang et al. 2020).

### Phylogenetic analysis

We used a concatenated set of base sequences of the 13 PCGs from 34 pigeons and doves to investigate the phylogenetic position of *T. curvirostra* (Table 2). Yellow-throated Sandgrouse (*Pterocles gutturalis* Smith, 1836) was used as an outgroup. All operations were performed in the PhyloSuite software package (Zhang et al. 2020). The sequences were aligned in batches using MAFFT software (Katoh et al. 2002). ModelFinder was used to partition the codons and identify the best substitution model for the phylogenetic analyses (Kalyaanamoorthy et al. 2017). Phylogenetic trees were constructed with Bayesian inference (BI) and maximum-likelihood (ML) (Yang 1994; Huelsenbeck and Ronquist 2001). The best substitution model of BI was selected according to codon 1, 2 and 3, while the model of ML was determined by the automatic partitioning (Table 3). For the BI tree, Markov chains were run for one million generations and were sampled every 100 generations. The majority-rule consensus trees were estimated by combining the results from duplicated analyses, while discarding the first 25% of generations. Besides, we checked for nuclear copies of mitochondrial sequences (numts) and possible chimerism (Sangster et al. 2016; Sangster and Luksenburg 2020).

**Table 2.** Summary of the mitogenomes used in the analyses.

Family	Subfamily	Genus	Species	Accession
Columbidae	Columbinae	* Geopelia *	*Geopelia cuneata*	MN930521.1
			*Geopelia striata*	MG590276.1
		* Trugon *	*Trugon terrestris*	MG590263.1
		* Caloenas *	*Caloenas maculata*	KX902249.1
			*Caloenas nicobarica*	MG590264.1
		* Streptopelia *	*Streptopelia tranquebarica*	MT535858
			*Streptopelia orientalis*	KY827037.1
			*Streptopelia decaocto*	KY827036.1
			*Streptopelia chinensis*	KP273832.1
		* Columba *	*Columba hodgsonii*	MN919176.1
			*Columba janthina*	LC541479.1
			*Columba jouyi*	KX902247.1
			*Columba livia*	KP319029.1
			*Columba rupestris*	KX902246.1
		* Ectopistes *	*Ectopistes migratorius*	KC489473.1
		* Patagioenas *	*Patagioenas fasciata*	KX902240.1
		* Leptotila *	*Leptotila verreauxi*	HM640214.1
		* Zenaida *	*Zenaida macroura*	KX902235.1
			*Zenaida auriculata*	HM640211.1
		* Geotrygon *	*Geotrygon violacea*	HM640213.1
		* Turtur *	*Turtur tympanistria*	HM746793.1
		* Columbina *	*Columbina picui*	MN356335.1
		* Chalcophaps *	*Chalcophaps indica*	HM746789.1
	Treroninae	* Alopecoenas *	*Alopecoenas salamonis*	KX902250.1
		* Hemiphaga *	*Hemiphaga novaeseelandiae*	EU725864.1
	Gourinae	* Goura *	*Goura cristata*	MG590273.1
			*Goura sclaterii*	MG590285.1
			*Goura scheepmakeri*	MG590282.1
			*Goura victoria*	MG590299.1
		* Pezophaps *	*Pezophaps solitaria*	KX902238.1
		* Raphus *	*Raphus cucullatus*	KX902236.1
	Otidiphabinae	* Otidiphaps *	*Otidiphaps nobilis*	MG590265.1
	Didunculinae	* Didunculus *	*Didunculus strigirostris*	MG590266.1
Pteroclidae		* Pterocles *	*Pterocles gutturalis*	MN356147.1

**Table 3.** The best substitution models for Bayesian inference (BI) and maximum-likelihood (ML) analyses.

		ND1	ND2	COI	COII	ATP6	ATP8	COIII	ND3	ND4L	ND4	ND5	Cyt b	ND6
BI	Codon 1	SYM + I + G4	GTR + F + I + G4	SYM + I + G4	SYM + I + G4	GTR + F + I + G4	GTR + F + G4	SYM + I + G4	SYM + I + G4	GTR + F + I + G4	GTR + F + I + G4	GTR + F + I + G4	SYM + I + G4	GTR + F + G4
	Codon 2	GTR + F + I + G4	GTR + F + I + G4	GTR + F + I + G4	GTR + F + I + G4	GTR + F + I + G4	GTR + F + I + G4	GTR + F + I + G4	GTR + F + I + G4	GTR + F + I + G4	GTR + F + I + G4	GTR + F + I + G4	GTR + F + I + G4	HKY + F + I + G4
	Codon 3	GTR + F + I + G4	GTR + F + I + G4	GTR + F + I + G4	GTR + F + I + G4	GTR + F + I + G4	GTR + F + I + G4	GTR + F + I + G4	GTR + F + I + G4	GTR + F + I + G4	GTR + F + I + G4	GTR + F + I + G4	GTR + F + I + G4	GTR + F + G4
ML		TVM + F + R4	TVM + F + R4	TIM2 + F + I + G4	TIM2 + F + I + G4	TVM + F + R4	TN + F + I + G4	TIM2 + F + I + G4	TIM2 + F + I + G4	TIM2 + F + I + G4	TVM + F + R4	TVM + F + R4	TIM2 + F + I + G4	TIM2 + F + I + G4

## Results and discussion

### Mitochondrial genome structure and organization

**Figure 1.**
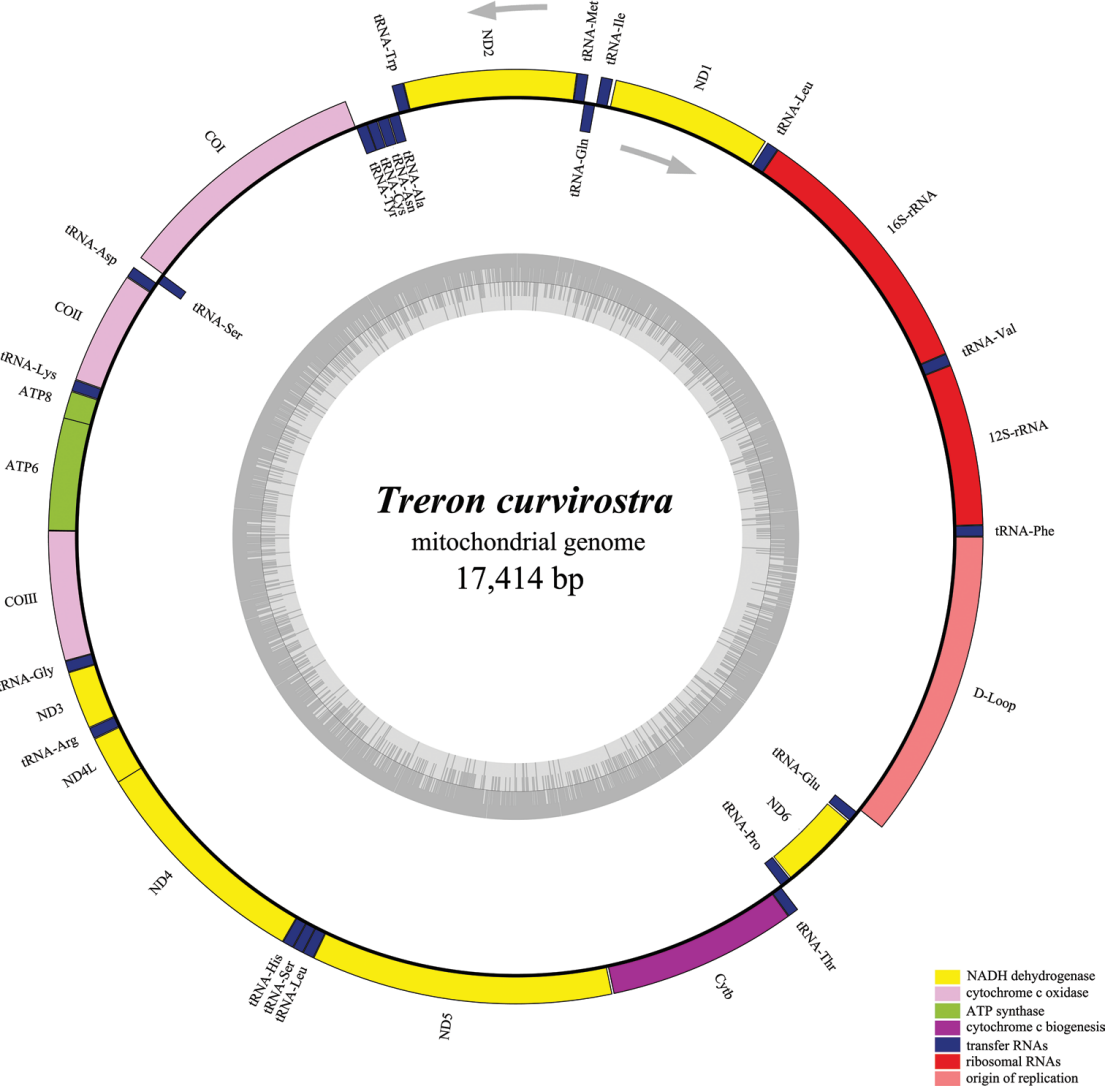
Circular map of the *T. curvirostra* mitochondrial genome.

The mitochondrial genome of the Thick-billed Green-Pigeon was found to be 17,414 bp in length, which agrees with the length of most of the other sequenced species of pigeons and doves (Table 4, Table 5, Table 6) (Pereira et al. 2007; Zhang et al. 2015). In addition, the base composition of *T. curvirostra* was found to be A = 30.32%, G = 13.61%, T = 24.83%, and C = 31.24%), where the A+T content (55.15%) was higher than the G+C content (44.85%) and is similar to other birds in Columbidae (Table 5 and Table 6) (Huang et al. 2016; Jang et al. 2016) . Moreover, the genome had a closed circular ring structure, containing 22 tRNAs, 2 rRNAs, 13 PCGs, and one D-loop. The ND6 gene and the other 8 tRNAs (tRNA-Gln, tRNA-Ala, tRNA-Asn, tRNA-Cys, tRNA-Tyr, tRNA-Ser (UGA), tRNA-Pro, and tRNA-Glu) were transcribed from the light (L)-strand, while the other genes were transcribed from the heavy (H)-strand (Fig. 1, Table 4). In addition, two pairs of overlapping regions among the ATP6/COIII and ND4L/ND4 were found, with an overlapping region of ATP6/COIII being one bp and the overlapping region of ND4L/ND4 being seven bp. Furthermore, 18 intergenic spacers were observed between the mitochondrial regions with lengths between -7 and 17 bp. Among all these intergenic spacers, the shortest was -7 bp (found between ND4L and ND4), while the longest was 17 bp (found between ND1 and tRNA-Ile).

**Table 4.** Mitochondrial genetic composition of *T. curvirostra*.

Gene	Strand	Position	Anticodon	Size (bp)	Start codon		Intergenic length
tRNA-Phe	H	1–68	GAA	68			0
12S rRNA	H	69–1041		973			0
tRNA-Val	H	1042–1114	UAC	73			0
16S rRNA	H	1115–2703		1589			0
tRNA-Leu	H	2704–2777	UAA	74			12
ND1	H	2790–3755		966	ATG	AGA	17
tRNA-Ile	H	3773–3844	GAU	72			5
tRNA-Gln	L	3850–3920	UUG	71			0
tRNA-Met	H	3921–3988	CAU	68			0
ND2	H	3989–5027		1039	ATG	T	0
tRNA-Trp	H	5028–5098	UCA	71			1
tRNA-Ala	L	5100–5168	UGC	69			2
tRNA-Asn	L	5171–5243	GUU	73			2
tRNA-Cys	L	5246–5313	GCA	68			0
tRNA-Tyr	L	5314–5384	GUA	71			1
COI	H	5386–6936		1551	ATG	AGG	0
tRNA-Ser	L	6937–7001	UGA	65			2
tRNA-Asp	H	7004–7072	GUC	69			1
COII	H	7074–7757		684	ATG	TAA	1
tRNA-Lys	H	7759–7828	UUU	70			1
ATP8	H	7830–7997		168	ATG	TAA	0
ATP6	H	7988–8671		684	ATG	TAA	-1
COIII	H	8671–9454		784	ATG	T	0
tRNA-Gly	H	9455–9523	UCC	69			0
ND3	H	9524–9875		352	ATT	TAA	1
tRNA-Arg	H	9877–9945	UCG	69			1
ND4L	H	9947–10243		297	ATG	TAA	-7
ND4	H	10237–11614		1378	ATG	T	0
tRNA-His	H	11615–11683	GUG	69			0
tRNA-Ser	H	11684–11749	GCU	66			0
tRNA-Leu	H	11750–11819	UAG	70			0
ND5	H	11820–13637		1818	ATG	AGA	8
Cyt b	H	13646–14788		1143	ATG	TAA	0
tRNA-Thr	H	14789–14856	UGU	68			6
tRNA-Pro	L	14863–14932	UGG	70			4
ND6	L	14937–15458		522	ATG	TAG	3
tRNA-Glu	L	15462–15532	UUC	71			0
D-loop		15553–17414		1862			0

### The PCGs

**Figure 2.**
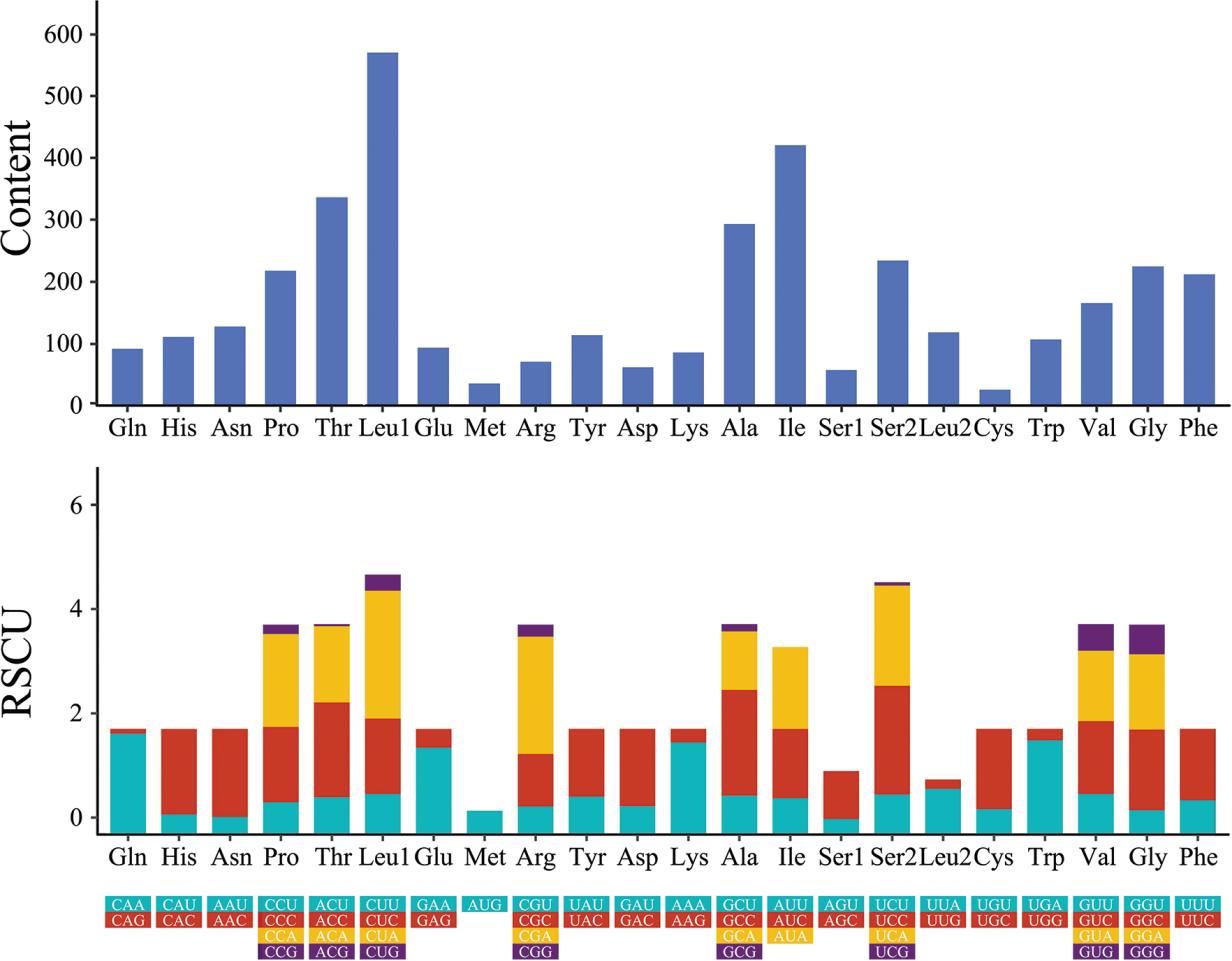
Codon distribution and relative synonymous codon usage in *T. curvirostra* mitogenome.

**Figure 3.**
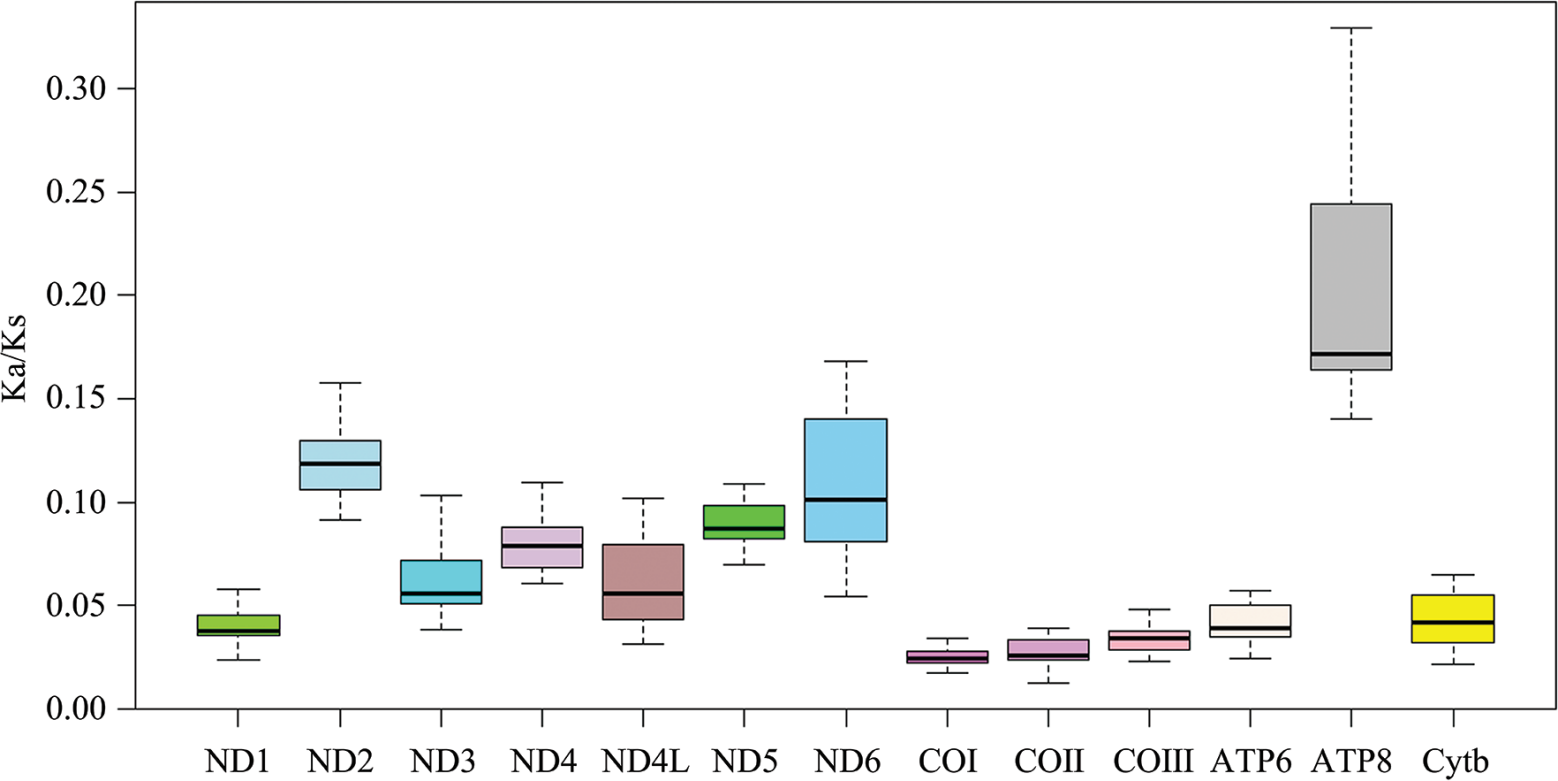
The ratio of the number of nonsynonymous substitutions per nonsynonymous site to the number of synonymous substitutions per synonymous site of 13 PCGs among 17 species of pigeons and doves. *T. curvirostra* was set as a baseline.

The total length of the PCGs was 11,386 bp, which is consistent with the average length of PCGs found in Columbidae (Table 5). The base composition of PCGs was A = 29.46%, G = 12.23%, T = 24.56%, and C = 33.76%, while the A+T content (54.01%) was slightly higher than the G+C content (45.99%). The AT-skew of *T. curvirostra* was positive, while the GC-skew was negative (Table 5). Furthermore, the PCG regions of *T. curvirostra* contained genes coding for cytochrome b (Cyt b), two ATPases (ATP6 and ATP8), three cytochrome c oxidases (COI, COII, and COIII), and seven NADH dehydrogenases (ND1-6 and ND4L). With the exception of ND3 (which had ATT as its start codon), all the other PCGs had ATG as a start codon. Six PCGs had the complete stop codon of TAA, while four PCGs had the other complete stop codons of AGA (ND1 and ND5), AGG (COI), and TAG (ND6). ND2, ND4, and COIII had the incomplete stop codon of T (Table 4). The RSCU of *T. curvirostra* is illustrated in Fig. 2, where Leu1 had the highest concentration and Cys had the lowest. In addition, Met only had AUG, while the other seven regions had four codons. With *T. curvirostra* as a baseline, the Ka/Ks ratio (Hurst 2002) of the 13 PCGs in 17 species of doves were all less than 1, with the highest Ka/Ks ratio (0.1937) in ATP8 and the lowest ratio (0.0243) in COI (Fig. 3). Hence, it seems that evolution tended to be conservative and maintained the generated protein (Hanada et al. 2007).

**Table 5.** Composition and skewness in mitochondrial genome of *T. curvirostra*.

Region	A%	T%	AT-skew	G%	C%	GC-skew
whole mitogenome	30.32	24.83	0.100	13.61	31.24	-0.393
PCGs	29.46	24.56	0.091	12.23	33.76	-0.468
rRNAs	32.75	21.19	0.214	19.05	27.01	-0.173
tRNAs	32.33	25.16	0.125	16.95	25.55	-0.203
D-loop	30.45	31.31	-0.014	11.92	26.32	-0.376

**Table 6.** Nucleotide composition indices in different regions of mitogenomes of Columbidae.

Species	GenBank no	Whole mitogenome		Protein coding genes		Ribosomal RNA	
		Length (bp)	AT (%)	Length (bp)	AT (%)	Length (bp)	AT (%)
*Goura sclaterii*	MG590285.1	18242	54.13	11386	52.99	2571	53.21
*Treron curvirostra*	MT535857	17414	55.16	11386	54.02	2562	53.94
*Columba hodgsonii*	MN919176.1	17477	54.55	11385	53.82	2557	53.23
*Trugon terrestris*	MG590263.1	17405	55.62	11395	54.94	2569	55.86
*Didunculus strigirostris*	MG590266.1	17389	54.94	11390	54.32	2569	55
*Geopelia striata*	MG590276.1	17354	54.83	11383	53.78	2565	54.07
*Otidiphaps nobilis*	MG590265.1	17346	55.83	11382	55.2	2581	54.75
*Hemiphaga novaeseelandiae*	EU725864.1	17264	54.89	11386	54.1	2575	54.52
*Caloenas nicobarica*	MG590264.1	17178	55.13	11386	54.83	2567	53.64
*Leptotila verreauxi*	HM640214.1	17176	54.1	11383	53.34	2556	53.4
*Alopecoenas salamonis*	KX902250.1	17141	55.18	11381	54.78	2569	54.81
*Streptopelia orientalis*	KY827037.1	17102	53.41	11386	52.86	2561	53.58
*Raphus cucullatus*	KX902236.1	17092	56.08	11385	55.87	2566	54.6
*Ectopistes migratorius*	KC489473.1	17026	54.52	11383	54.3	2596	52.97
*Patagioenas fasciata*	KX902239.1	16970	54.51	11384	54.02	2550	53.8
*Geotrygon violacea*	HM640213.1	16864	55.04	11383	54.27	2561	53.85
*Zenaida auriculata*	HM640211.1	16781	53.29	11380	52.71	2567	52.83

### Transfer RNAs, ribosomal RNAs, and the D-loop

**Figure 4.**
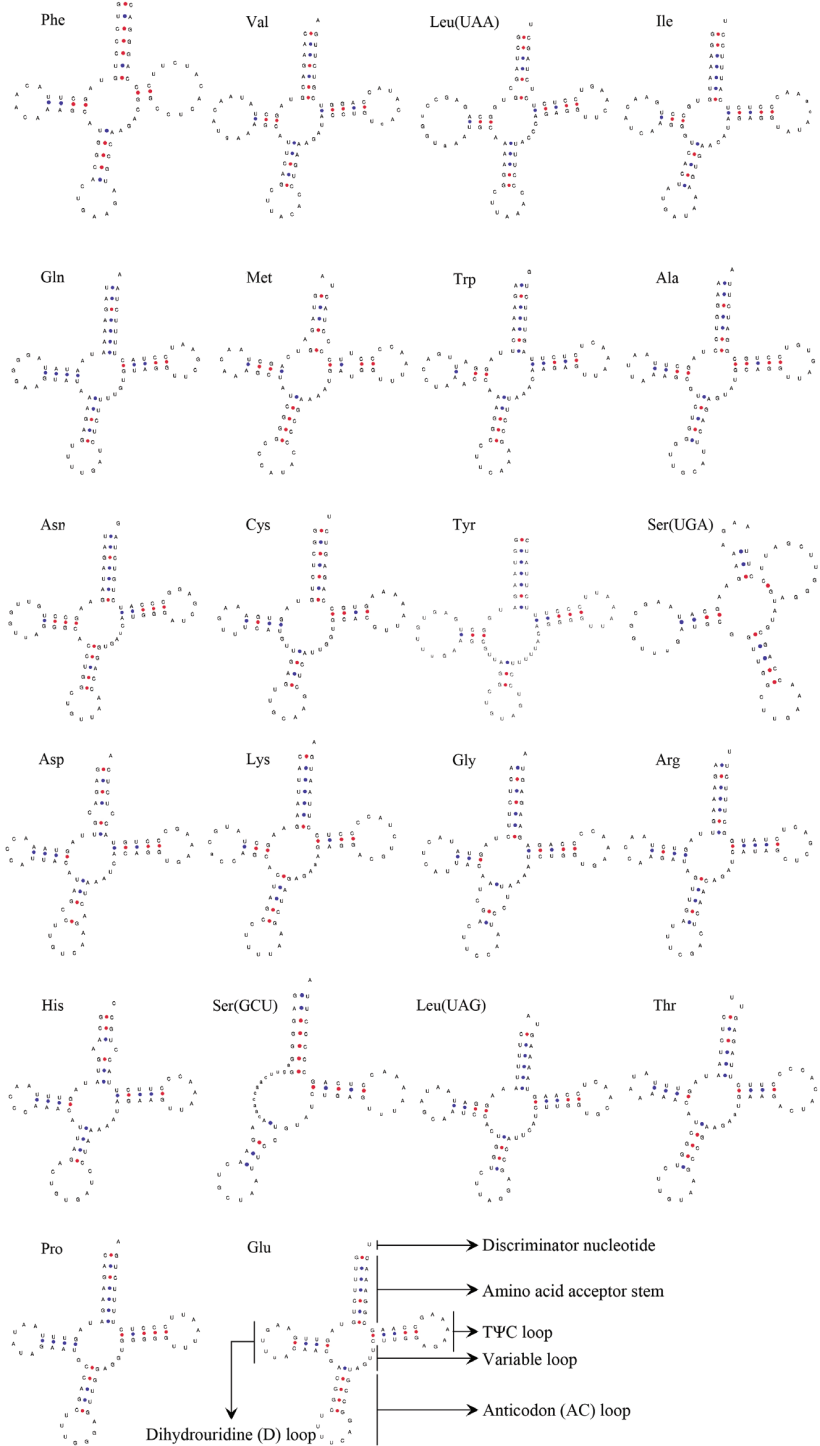
Secondary structure of 22 tRNA genes from the *T. curvirostra* mitochondrial genome.

The mitogenome of *T. curvirostra* contained 22 tRNAs with lengths ranging from 65 bp (tRNA-Ser (UGA)) to 74 bp (tRNA-Leu (UAA)), which is similar to that in the mitogenomes of other pigeons and doves (Zhang et al. 2015). Moreover, the total length of the tRNAs was 1,534 bp, with an A+T content of 57.50%, a G+C content of 42.50%, an AT-skew of 0.1247, and a GC-skew of -0.2025 (Table 5). Among all the secondary structures of the 22 tRNA genes from the *T. curvirostra* mitochondrial genome, with the exception of tRNA-Ser (GCU), all had a typical cloverleaf structure (Fig. 4).

The total size of the two rRNAs was 2,562 bp, with an A+T content of 53.94%, an AT-skew of 0.2142, and a GC-skew of -0.1729 (Table 5). The 12S rRNA was 973 bp in length and was located between tRNA-Phe and tRNA-Val, while the 16S rRNA was 1,589 bp in length and was located between tRNA-Val and tRNA-Leu (UAA).

A D-loop was found between tRNA-Glu and tRNA-Phe, and was 1,862 bp in length, with a A+T content of 61.76%, an AT-skew of -0.0139, and a GC-skew of -0.3764 (Table 5). Duplication and rearrangement of the avian mitochondrial genomes is common, but *T. curvirostra* had only one D-loop, which is similar to that present in other known mitogenomes of Columbidae (Pacheco et al. 2011; Eberhard and Wright 2016; Bruxaux et al. 2018).

### Phylogenetic analysis

**Figure 5.**
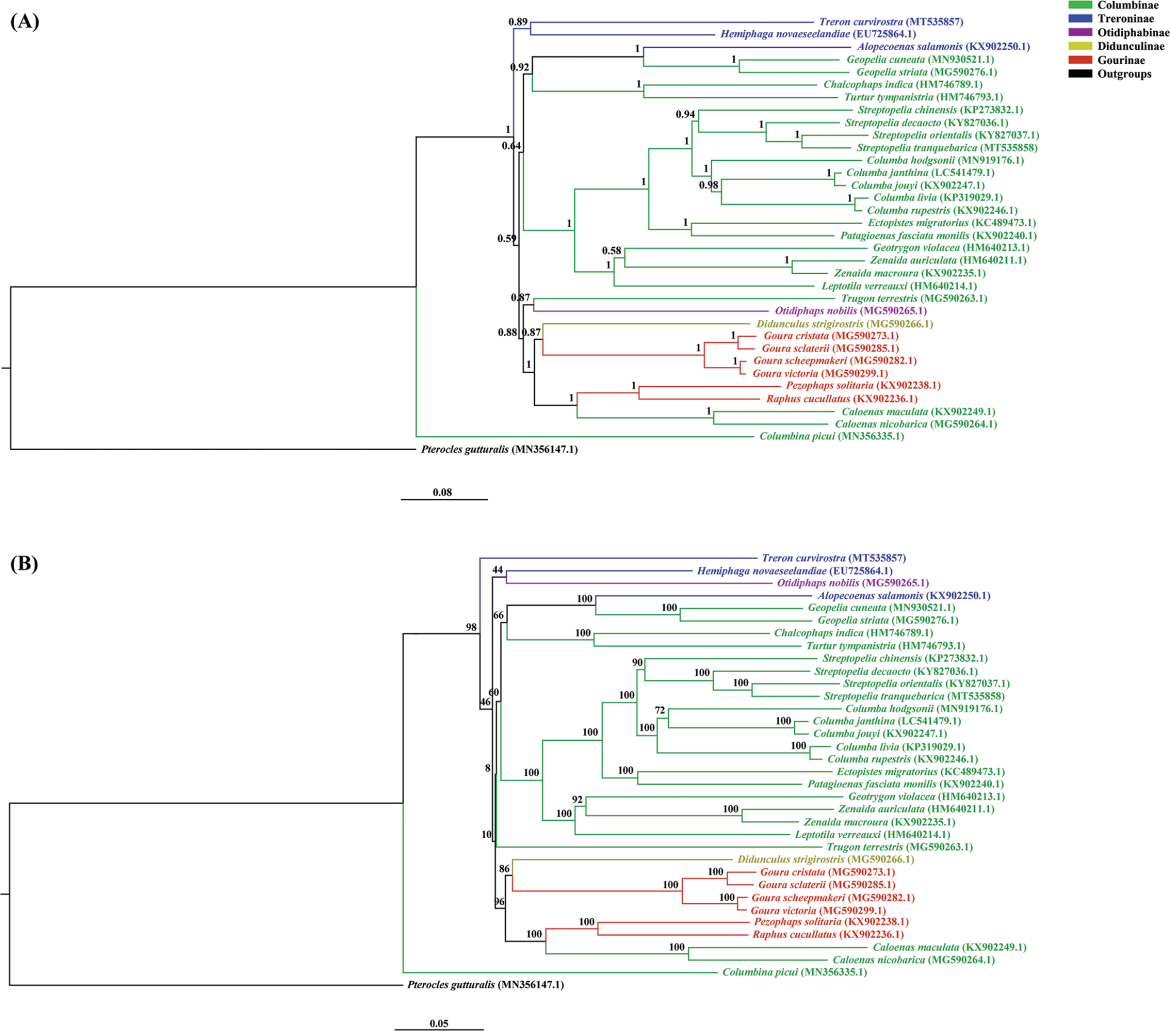
Mitogenomic phylogeny of 34 Columbidae species and an outgroup (*Pterocles gutturalis*) based on 13 PCGs using the Bayesian inference (**A**) and maximum likelihood (**B**) methods. Different colors indicated different subfamilies.

Although the topology of ML tree and BI tree were similar to each other, they differed with respect to the phylogenetic position of *T. curvirostra*. *Treron curvirostra* clustered with *Hemiphaga novaeseelandiae* (Gmelin, 1789) in the BI tree, whereas it did not cluster with any species in the ML tree (Fig. 5). Therefore, we tested for the presence of the numts and chimerism. All these tests were negative, indicating the validity of *T. curvirostra* mitogenome. The phylogenetic trees also highlighted the stable relationships among the same genera within Columbidae, which was consistent with previous studies from analyses of mitochondrial and nuclear genes (Kan et al. 2010; Pacheco et al. 2011; Hung et al. 2013; Mlíkovský 2016; Soares et al. 2016; Kretschmer et al. 2020; Liu et al. 2020) (Fig. 5). However, the phylogenetic analysis did not support the arrangement of pigeons into five subfamilies (Columbinae, Didunculinae, Gourinae, Otidiphabinae, and Treroninae) as recognized by ITIS. *Caloenas*, *Geopelia*, and *Trugon terrestris* (which were placed in Columbinae by ITIS) clustered with species from other subfamilies in our phylogenies (Fig, 5). The most likely cause might be that the original classification system was based mainly on patterns of overall similarity in morphology which may not accurately reflect phylogenetic relationships. Similar contradictions between overall similarity and phylogeny have also been found in other groups of birds, including terns (Bridge et al. 2005), rails (Sangster et al. 2015), nightjars (Han et al. 2010), eagles (Lerner and Mindell 2005), laughing thrushes (Luo et al. 2008), and chats and flycatchers (Sangster et al. 2010). Our results indicate that the subfamily classification of Columbidae may not accurately reflect historical relationships and may need to be revised. However, the poor branch support of basal clades of Columbidae precludes such a revision at present. Clearly, future attempts to resolve the phylogeny of Columbidae with confidence should include a suitable set of nuclear markers.
